# Synthetic Pinnatoxins A and G Reversibly Block Mouse Skeletal Neuromuscular Transmission In Vivo and In Vitro

**DOI:** 10.3390/md17050306

**Published:** 2019-05-24

**Authors:** Evelyne Benoit, Aurélie Couesnon, Jiri Lindovsky, Bogdan I. Iorga, Rómulo Aráoz, Denis Servent, Armen Zakarian, Jordi Molgó

**Affiliations:** 1 Commissariat à l’Energie Atomique et aux énergies Alternatives (CEA), Institut des Sciences du Vivant Frédéric Joliot, Service d’Ingénierie Moléculaire des Protéines (SIMOPRO), CEA de Saclay, Université Paris-Saclay, F-91191 Gif-sur-Yvette, France; evelyne.benoit@cea.fr (E.B.); romulo.araoz@cea.fr (R.A.); denis.servent@cea.fr (D.S.); 2 Centre National de la Recherche Scientifique (CNRS), Institut des Neurosciences Paris-Saclay (Neuro-PSI), UMR 9197 CNRS/Université Paris-Sud, F-91198 Gif-sur-Yvette, France; aurelie.couesnon@inserm.fr (A.C.); jiri.lindovsky@img.cas.cz (J.L.); 3 Centre National de la Recherche Scientifique (CNRS), Institut de Chimie des Substances Naturelles, UPR 2301, Labex LERMIT, F-91198 Gif-sur-Yvette, France; bogdan.iorga@cnrs.fr; 4 Department of Chemistry and Biochemistry, University of California, Santa Barbara, CA 93106, USA; zakarian@chem.ucsb.edu

**Keywords:** pinnatoxins, marine phycotoxins, mouse neuromuscular system, compound muscle action potential, synaptic potentials, emerging toxins, cyclic imines

## Abstract

Pinnatoxins (PnTXs) A-H constitute an emerging family belonging to the cyclic imine group of phycotoxins. Interest has been focused on these fast-acting and highly-potent toxins because they are widely found in contaminated shellfish. Despite their highly complex molecular structure, PnTXs have been chemically synthetized and demonstrated to act on various nicotinic acetylcholine receptor (nAChR) subtypes. In the present work, PnTX-A, PnTX-G and analogue, obtained by chemical synthesis with a high degree of purity (>98%), have been studied in vivo and in vitro on adult mouse and isolated nerve-muscle preparations expressing the mature muscle-type (α1)_2_β1δε nAChR. The results show that PnTX-A and G acted on the neuromuscular system of anesthetized mice and blocked the compound muscle action potential (CMAP) in a dose- and time-dependent manner, using a minimally invasive electrophysiological method. The CMAP block produced by both toxins in vivo was reversible within 6–8 h. PnTX-A and G, applied to isolated extensor digitorum longus nerve-muscle preparations, blocked reversibly isometric twitches evoked by nerve stimulation. The action of PnTX-A was reversed by 3,4-diaminopyridine. Both toxins exerted no direct action on muscle fibers, as revealed by direct muscle stimulation. PnTX-A and G blocked synaptic transmission at mouse neuromuscular junctions and PnTX-A amino ketone analogue (containing an open form of the imine ring) had no effect on neuromuscular transmission. These results indicate the importance of the cyclic imine for interacting with the adult mammalian muscle-type nAChR. Modeling and docking studies revealed molecular determinants responsible for the interaction of PnTXs with the muscle-type nAChR.

## 1. Introduction

Pinnatoxins (PnTXs) A–D were originally identified in the digestive glands of the bivalve mollusks *Pinna attenuata* and *Pinna muricata* following food poisoning outbreaks that were linked to these shellfish in China and Japan [1,2,3,4]. However, it is still unclear whether PnTXs were the cause of these poisoning events. Later, three new PnTX-A analogues, the PnTX-E, F, and G, were isolated and structurally characterized from extracts of Pacific oysters (*Crassostrea gigas*) and razorfish (*Pinna bicolor*) from South Australia [5,6].

Analysis of surface sediments in New Zealand and South Australia, where shellfish were reported positive for PnTXs, led to the discovery of a peridinoid dinoflagellate producing PnTX-E and F in New Zealand, PnTX-E, F, and G in South Australia [7,8,9] and PnTX-G in Japan [10]. Morphological and phylogenetic similarities were observed between these dinoflagellates and a new dinoflagellate named *Vulcanodinium rugosum* discovered in water samples of Mediterranean lagoons in the French coast [11]. Contamination of mussels and clams by PnTXs, and the link to the *V. rugosum* dinoflagellate was first reported in France in 2011 [12], but retro-analysis of contaminated shellfish samples revealed high levels of PnTX-G since 2006 [13]. Likewise, PnTXs have been found in other European waters and seafood since 2010 [14,15,16,17,18], and in Canada as well [19]. There is also evidence that the harmful *V. rugosum* dinoflagellate can be transported in ballast tanks of shipping vessels [20], which is of global concern. Also, new strains of the *V. rugosum* dinoflagellate, isolated from the South China Sea [21] and the Arabian Gulf [22], were reported to produce only PnTX-H and portimine [21,23], as determined by liquid chromatography-tandem mass spectrometry (LC-MS/MS).

**Figure 1:**
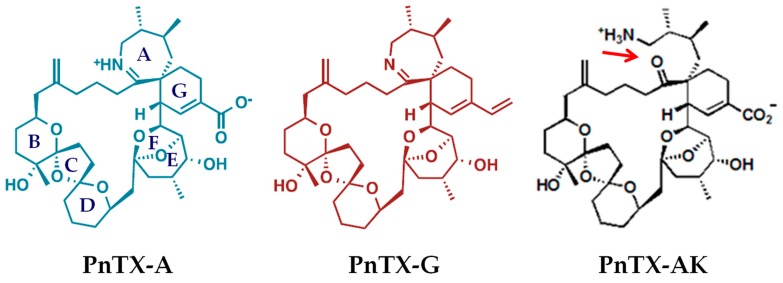
Chemical structures of PnTX-A, PnTX-G and PnTX-A amino ketone analogue (PnTX-AK).

PnTXs belong to a heterogeneous and growing group of macrocyclic compounds called “cyclic imines toxins” that include the prorocentrolides, spiro-prorocentrimine, gymnodimines, spirolides, pteriatoxins, and portimines ([24,25,26] for reviews, and [27,28] for recently described cyclic imine toxins). Up until now, eight PnTXs (A–H) have been reported. Their chemical structure contains a common scaffold characterized by a dimethyl substituted 7-membered cyclic imine as part of a spiroimine ring system, a 6,5,6-spiroketal ring system, and a bridged ketal which is typical of this family of toxins [24,25,26], as exemplified for PnTX-A and G in Figure 1. It has been proposed that PnTX-F and G are the precursors of all PnTXs, as well as of the structurally related pteriatoxins, via metabolic and hydrolytic transformations in shellfish [6]. Interestingly, in contrast to other cyclic imine toxins, PnTXs exhibit an outstanding chemical stability at acid pH (pH 1.5 and pH 4.0) [6,29].

In mouse bioassays, PnTx-E, F, and G were shown to produce rapid lethality by respiratory depression upon intraperitoneal administration, with both neurological symptoms and skeletal muscle flaccid paralysis [6,23,30]. Among the cyclic imine phycotoxins purified, PnTX-E, F, and G were the ones that exhibited the highest acute oral mouse toxicity [30].

PnTXs, like other cyclic imine toxins, are known to be potent antagonists of both *Torpedo* muscle-type (α1_2_β1γδ) and neuronal α7, α4β2 and α3β2 nicotinic acetylcholine receptors (nAChRs) [31,32,33]. Studies on isolated rat phrenic-hemidiaphragm preparations showed that crude extract containing a mixture of PnTX-E and PnTX-F, as well as purified PnTX-F [34] and purified PnTX-E, PnTX-F, and PnTX-G [35] produced concentration-dependent decreases in nerve-evoked muscle twitches with a rank order of potency of PnTX-F > PnTX-G > PnTX-E, incomplete washout profiles for PnTX-F and PnTX-G, and the inability to be reversed by the anticholinesterase inhibitor neostigmine [34,35].

To the best of our knowledge, neither PnTX-A nor PnTX-G, obtained by chemical synthesis and having an established degree of purity (>98%), have been studied in vivo and in vitro on adult mouse and isolated nerve-muscle preparations expressing the mature muscle α1_2_β1δε nAChR. Therefore, aims of the current study were (i) to study the local action of synthetic PnTX-A and G on the neuromuscular system of anesthetized mice in vivo, using a minimally invasive electrophysiological method, (ii) to study in vitro the actions of PnTX-A and G, as well as PnTX-A amino ketone derivative (PnTX-AK, containing an open form of the imine ring), on isometric twitch tension and on cholinergic transmission at single neuromuscular junctions, (iii) to determine the eventual efficacy of 3,4-diaminopyridine for reversing the neuromuscular blockade produced by PnTX-A and G, and (iv) to model the molecular interactions between PnTX-A and G and the α1_2_β1δε nAChR.

## 2. Results

### 2.1. Effects of PnTX-A and G on the Excitability Properties of Mouse Neuromuscular System In Vivo

**Figure 2:**
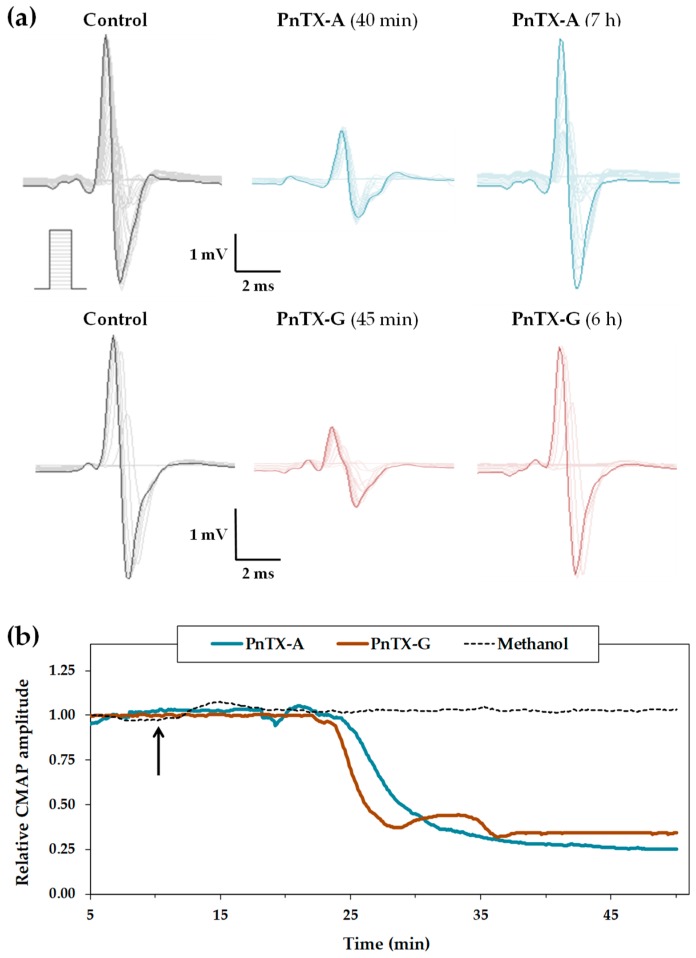
Effects of local injections of PnTX-A and G on the multimodal excitability properties of mouse neuromuscular system in vivo. (**a**) Traces of CMAP recorded from the tail muscle following increasing intensities of caudal motor nerve stimulation (scheme), before (control) and after injection of PnTX-A (5.44 nmol/kg of mouse, upper traces) or PnTX-G (3.20 nmol/kg of mouse, lower traces). (**b**) On-line recordings of the effects of PnTX-A (5.44 nmol/kg of mouse), PnTX-G (3.20 nmol/kg of mouse) and/or methanol (1%) injections on the CMAP maximal amplitude registered continuously over time. Values are expressed relatively to those before injections. The arrow indicates the time of injections.

The effects of intramuscular injections of PnTX-A and G were studied on the multimodal excitability properties of the mouse neuromuscular system in vivo (Figure 2).

On-line recordings were initiated approximately 10 min before a given injection of PBS containing methanol (0.1–1%) and various doses of either PnTX-A (from 0.54 to 5.44 nmol/kg of mouse) or PnTX-G (1.60 and 3.20 nmol/kg of mouse), to observe the alterations occurring on some selected excitability parameters, such as the CMAP amplitude and excitability threshold, registered continuously over time. The major effect of the two toxins was a decrease of CMAP amplitude, as exemplified in Figure 2a,b for 5.44 nmol of PnTX-A per kg of mouse and 3.20 nmol of PnTX-G per kg of mouse. This effect occurred at various times, depending on the dose of toxin injected [PnTX-A: from 43.3 ± 0.2 min (n = 3 mice) for 0.54 nmol/kg of mouse to 26.1 ± 7.7 min (n = 4 mice) for 5.44 nmol/kg of mouse, and PnTX-G: from 24.4 ± 9.7 min (n = 4 mice) for 1.60 nmol/kg of mouse to 15.5 ± 7.0 min (n = 4 mice) for 3.20 nmol/kg of mouse]. The toxin-induced decrease of CMAP amplitude was completely reversed within 6-8 h after PnTX-A (5.44 nmol/kg of mouse) or PnTX-G (3.20 nmol/kg of mouse) injection, leading to a 93.7 ± 8.0% (n = 8 mice) recovery compared to control conditions, i.e., CMAP amplitude before injection (Figure 2a). Finally, the CMAP amplitude remained stable before toxin injections, or before and after injection (5 µL) of PBS added only with 1% methanol (Figure 2b). Under these latter conditions, the CMAP maximal amplitude was not significantly affected (*P* = 0.332), i.e., 95.0 ± 5.6% within 57.7 ± 13.3 min (n = 3 mice), compared to control conditions. This indicates that injections of the vehicle associated to the highest doses of toxin studied had no significant effect on the CMAP amplitude.

**Figure 3:**
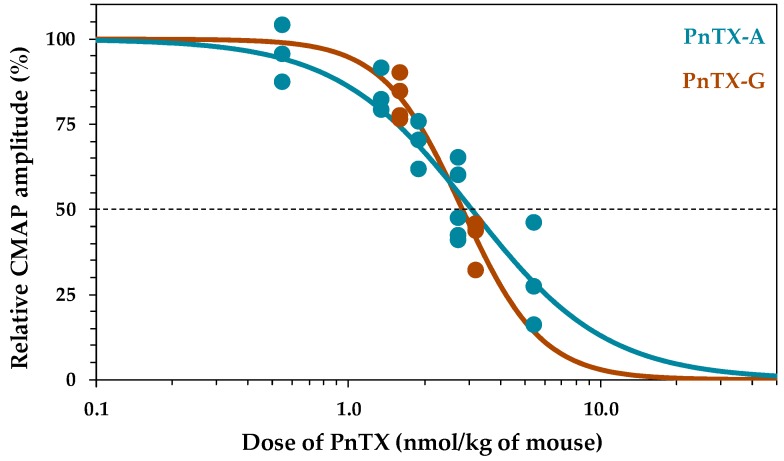
Dose-response curves of the effects of PnTX-A and G determined from CMAP maximal amplitude values recorded from the mouse tail muscle in vivo. Each value is expressed relatively to that obtained before injections. The curves were calculated from typical sigmoid non-linear regression through data points (r^2^ = 0.955 and 0.875 for PnTX-A and G, respectively). The dose required to block 50% of the CMAP maximal amplitude (ID_50_) and nH were, respectively, 3.1 ± 0.2 nmol/kg of mouse and 1.6 ± 0.2 (n = 18 mice) for PnTX-A, and 2.8 ± 0.1 nmol/kg of mouse and 2.7 ± 0.3 (n = 8 mice) for PnTX-G.

The dose-response curves of PnTX-A and G effects on the CMAP maximal amplitude were established and analyzed using individual values instead of mean values, for a better comparison of the efficacy between the two toxins. Results reveal that the doses necessary to inhibit 50% of the response (ID_50_) was 3.1 ± 0.2 (n = 18 mice) and 2.8 ± 0.1 (n = 8 mice) nmol/kg of mouse, respectively (Figure 3). These two ID_50_ values were not significantly different (*P* = 0.123). The comparison of PnTX-A and G ID_50_ values to those of some other cyclic imine toxins previously studied, i.e., gymnodimine-A (GYM-A) [36], 13-desmethyl spirolide C (13-SPX-C) [36] and 20-methyl spirolide G (20-meSPX-G) [37], shows that PnTX-A and G were as potent as GYM-A, but at least 300-fold less efficient than 13-SPX-C and 20-meSPX-G, to inhibit the CMAP maximal amplitude (Table 1).

The five different excitability tests (stimulus-response, strength-duration and current-threshold relationships, as well as threshold electrotonus and recovery cycle), performed together before and approximately 45 min after injections of PBS containing methanol (1%) and either PnTX-A (5.44 nmol/kg of mouse) or PnTX-G (3.20 nmol/kg of mouse) did not reveal further effects of the two toxins since no apparent modification of excitability waveforms was detected (data not shown). This was confirmed by analyzing the parameters determined from excitability tests, as exemplified in Table 2 for PnTX-A, with the exception of a significant lower “TEd (undershoot)” after PnTX-A-injection. Although this result may be due to alterations in slow voltage-gated K^+^ channels, it was not confirmed by analyzing the other parameters (such as “TEd (40–60 ms)”, “TEd (90–100 ms)” and “TEd (accommodation)”) related to this type of ion channel.

### 2.2. Effects of PnTX-A and G on Isometric Twitch Tension In Vitro

**Figure 4:**
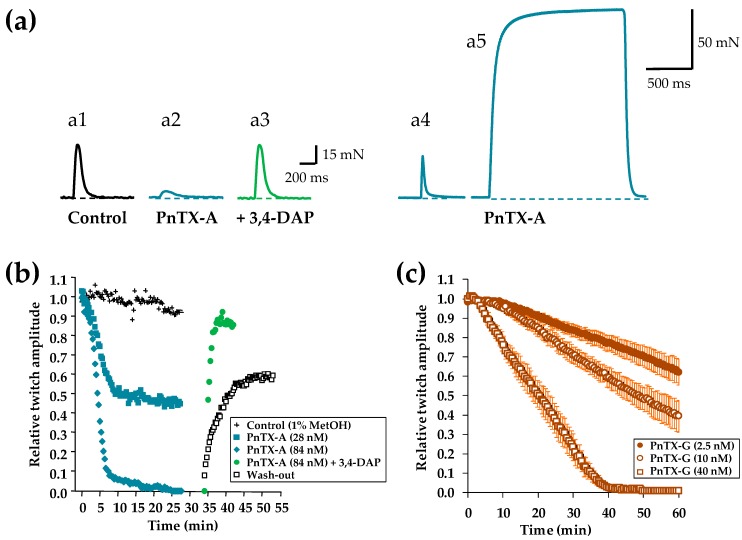
PnTX-A blockade of nerve-evoked isometric twitch tension without modification of directly elicited twitch and tetanus tension on isolated mouse EDL muscles. (**a**) (a1) Single twitch tension recording under control conditions, (a2) Marked reduction in twitch amplitude during the action of PnTX-A (54 nM), (a3) Reversal of the blockade produced by PnTX-A by 3,4-DAP (100 µM). (a4,a5) Twitch and tetanus responses evoked by direct muscle stimulation at 0.03 and 80 HZ, respectively in an EDL muscle in which nerve-evoked contractions were completely blocked by 56 nM PnTX-A. (**b**,**c**) Time course and concentration dependence of PnTX-A and PnTX G effects on nerve-evoked twitch responses, and the reversal by wash-out and 3,4-DAP (100 µM). After an equilibration period of 20 min PnTXs were applied at time 0. In (**b**) the wash-out of PnTX-A (84 nM), and the fast reversal of PnTX-A action by 3,4-DAP are shown. Note the slower onset kinetics of PnTX-G (**c**) as compared to PnTX-A (**b**) on nerve-evoked muscle contraction. Each value is expressed relatively to that obtained before addition of PnTXs.

PnTX-A (2.8–84 nM) and PnTX-G (2.5–40 nM) when applied to isolated peroneal nerve-extensor digitorum longus (EDL) muscle preparations produced a concentration- and time-dependent reduction of the peak force amplitude of muscle twitches evoked by nerve stimulation at 0.03 Hz. Representative twitch-tension recordings are shown in Figure 4a. The onset of the neuromuscular block was fast with the higher concentration of PnTX-A used (84 nM), reaching 50% block in 5.1 ± 0.85 min (n = 3), and 100% block in 16.5 ± 1.5 min (n = 3). The time course of PnTX-A and PnTX-G actions on the relative isometric twitch amplitude are shown in Figure 4a,b. Although PnTX-G was more active than PnTX-A on nerve-evoked twitch responses, onset of the block was about two-times slower with PnTX-G as compared to PnTX-A (Figure 4b,c). Further experiments are needed for an understanding of the slower onset kinetics but higher potency of PnTX-G compared to PnTX-A. Complete and rapid reversal of PnTX-A induced neuromuscular block was obtained by addition of 100 µM 3,4-diaminopyridine (3,4-DAP) to the medium (Figure 4a,b).

**Figure 5:**
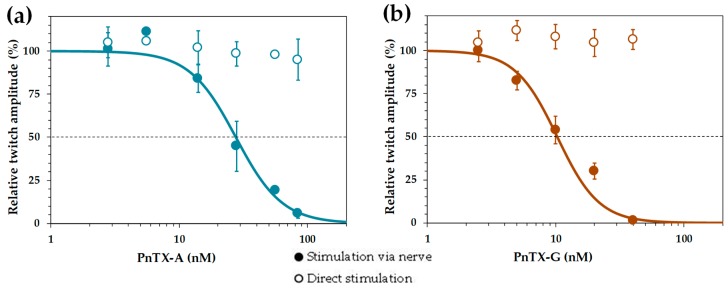
Concentration-response curves for PnTX-A (**a**) and PnTX-G (**b**) actions on the isometric twitch responses evoked by nerve stimulation (closed circles) or by direct muscle stimulation (open circles). Data points represent the mean ± S.D. of twitch response, after 60 min toxin exposure, relative to the respective controls of 3–6 EDL nerve-muscle preparations. The curves were calculated from typical sigmoid non-linear regression through data points (r^2^ = 0.989 and 0.922 for PnTX-A and G, respectively). The dose required to block 50% of the twitch amplitude (IC_50_) and nH were, respectively, 27.7 nM and 2.4 for PnTX-A, and 11.3 and 2.7 for PnTX-G.

The relative potencies of PnTX-A and G were evaluated by concentration-response curves. Calculation of the mean inhibitory concentration producing 50% reduction (IC_50_) of the isometric twitch response was of 27.7 nM for PnTX-A and 11.3 nM for PnTX-G (Figure 5a,b). The action of PnTX-A was reversible by washing out the toxin from the bathing medium within 120 min. Interestingly, in EDL muscles in which full block of nerve-evoked contraction was produced by either PnTX-A or PnTX-G, direct electrical muscle stimulation could still evoke twitch and tetanic contractions in response to single and tetanic direct muscle stimulation, respectively. A representative example is shown in Figure 4 (a4,a5). These results indicate that both PnTX-A and G exerted no direct action on the contractile machinery of muscle fibers, but acted on neuromuscular transmission, as previously shown for other cyclic imine toxins [37,38,39].

### 2.3. Effects of PnTX-A and G on Neuromuscular Transmission In Vitro

**Figure 6:**
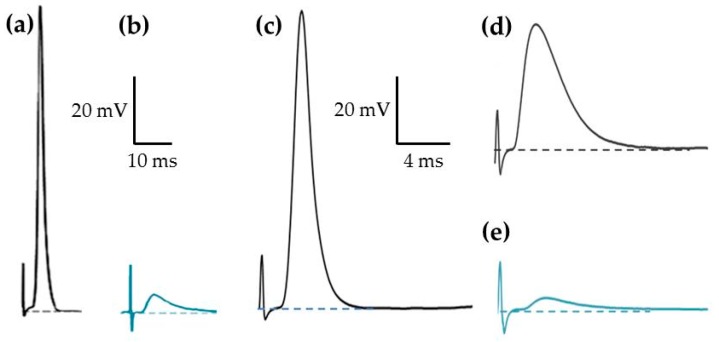
PnTX-A and G block skeletal neuromuscular transmission. (**a**) Nerve-evoked muscle action potential recorded in a junction of the EDL muscle upon nerve stimulation. (**b**) EPP recorded 30 min after the addition of PnTX-A (84 nM) to the standard Krebs-Ringer solution. (**c**) Muscle action potential recorded with a “floating” microelectrode in the FDB muscle upon nerve stimulation. (**d**) Full-sized EPP recorded after treatment with µ-conotoxin GIIIB (1.6 µM) in a junction of the FDB muscle. **(e)** EPP of reduced amplitude recorded after the action of PnTX-A. Recordings in (**a**) and (**b**) were obtained at a resting membrane potential of −70 mV, and those in (**c**) and (**d**,**e**) were obtained at −68 and −71 mV, respectively.

To evaluate the action of PnTXs on synaptic transmission, intracellular recordings at single neuromuscular junctions were performed from isolated EDL or FDB muscles. Experiments were first done in standard Krebs-Ringer solution containing physiological Ca^2+^ concentration (2 mM). Intracellular recordings at junctional regions of muscle fibers revealed that single nerve stimulation always triggered an action potential that sometimes dislodged the microelectrode due to the contraction of muscle fibers (Figure 6a). Muscle action potential triggered by nerve stimulation had overshoots of 21.3 ± 1.61 mV when recorded at mean resting membrane potential of −69.3 ±.1.06 mV (n = 16, 4 different EDL muscles). As shown in Figure 6b, addition of PnTX-A (84 nM) to the standard physiological medium completely abolished, within 30 min, the generation of muscle action potentials upon nerve stimulation. In addition, by recordings in junctional areas, these experiments revealed the presence of end-plate potentials (EPPs) of 3–5 mV amplitude that were unable to reach the threshold for action potential generation in muscle fibers. Similarly, when using a “floating” microelectrode, it was possible to record muscle action potentials in FDB muscles without movement-bias due to contraction (Figure 6c). Action potentials recorded under this condition were not significantly different from those successfully recorded with conventional microelectrodes (*P* < 0.05, n = 9).

Additional experiments were performed using µ-conotoxin GIIIB (1.0–1.6 µM) which blocks specifically and irreversibly voltage-gated skeletal muscle Na^+^ channels [40]. Under this condition, full-sized EPPs could be evoked by nerve stimulation without contraction, as shown in a representative recording in Figure 6d. Further addition of PnTX-A markedly reduced the amplitude of the full-sized EPPs (Figure 6e).

**Figure 7:**
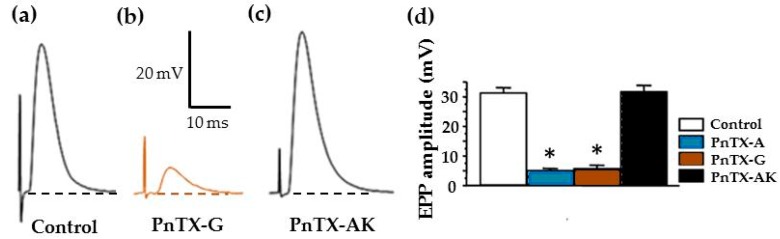
PnTX-A (84 nM) and PnTX-G (54 nM) block full-sized EPPs evoked by nerve stimulation, while 100 nM PnTX-AK (containing an open form of the imine ring) had no action on EPP amplitudes. (**a**) Representative control EPP recorded in a junction from an EDL muscle that has been treated with µ-conotoxin GIIIB to prevent muscle action potentials generation. (**b**) EPP recorded during the action of PnTX-G (20 nM). (**c**) Full-sized EPP recorded after 30 min of 100 nM PnTX-AK. (**d**) Graphs showing control EPP values (mean ± SEM; n = 4–8 for each condition), the significant (*: *P* < 0.05) reduction of EPP amplitudes by PnTX-A and G, and the lack of action of PnTX-AK (100 nM).

Both PnTX-A and G significantly blocked (*P* < 0.05) by more than 80% full-sized EPP amplitudes, recorded in muscles treated with µ-conotoxin GIIIB as shown in Figure 7. These results indicate that these toxins block neuromuscular transmission by reducing the amplitude of EPPs. It is worth noting that no significant change on the resting membrane potential of muscle fibers was detected in the presence of PnTX-A and G in the range of concentrations studied (*P* > 0.05; n = 12 and n = 9, respectively).

**Figure 8:**
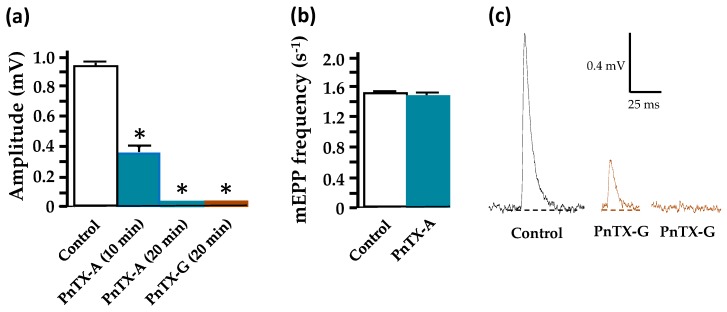
PnTX-A and PnTX-G block of mEPP amplitude without affecting mEPP frequency. (**a**) Control mEPP amplitude (white column), after 10 min and 20 min of 5 nM PnTX-A action (blue columns), and 5 nM PnTX-G action (brown column). (**b**) Frequency of mEPP recorded under control conditions (white column) and after 10 min of 5 nM PnTX-A action (blue column) when mEPPs of reduced amplitude were still present. Note, under this condition, the lack of action of PnTX-A on mEPP frequency. (**c**) Examples of mEPPs recorded under control conditions and during the action of 5 nM PnTX-G (brown traces). *: denotes significant differences compared to controls (*P* < 0.05).

Further experiments were performed to determine whether miniature end-plate potentials (mEPPs), which result from the release of a single quantum of ACh, are modified in amplitude and frequency by the PnTXs. Under control conditions, the mean amplitude of mEPPs recorded in standard Krebs-Ringer solution in unstimulated junctions from the EDL muscles was 0.92 ± 0.03 mV (n = 6, from 6 different muscles), addition of 5 nM PnTX-A or G to the medium induced a significant reduction and complete block of mEPP amplitude within 25 min with both toxins (n = 3, from 3 different muscles in each condition). The block of mEPP amplitude was fully reversible upon washing (within 30 min) of the nerve-muscle preparations with a toxin-free medium. Estimations of the frequency of mEPPs when mEPP were blocked by about 50%, but still easily recognized from the basal electric noise, revealed that the frequency was not significantly modified (Figure 8b).

Overall, our results show that synaptic transmission is strongly impaired by both PnTX-A and G, and suggest that both phycotoxins block, in a reversible manner, the interaction of ACh quanta with endplate nAChR.

**Figure 9:**
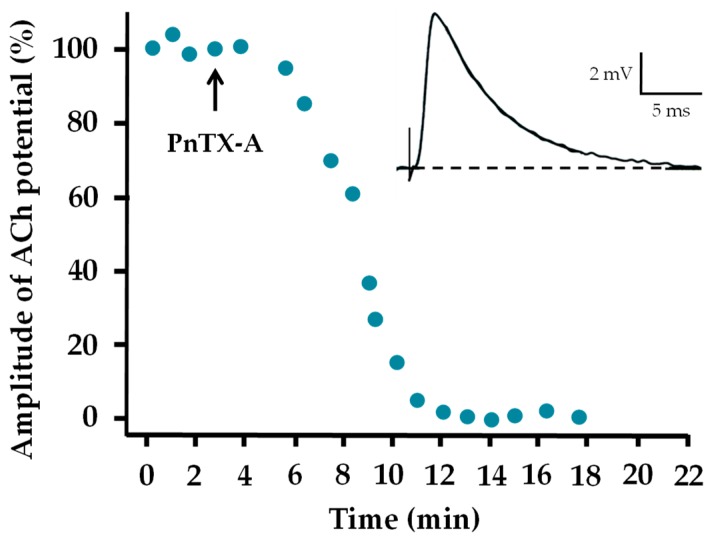
Time course of the block of ACh-evoked potentials by 100 nM PnTX-A applied to a superficial EDL neuromuscular junction. Constant iontophoretic ACh pulses of 1 ms duration were delivered to the endplate region (detected by an intracellular microelectrode through mEPP recordings) by a high resistance pipette containing 1 M ACh hydrochloride. Inset: typical control ACh-response obtained before the addition of PnTX-A to the standard Krebs-Ringer solution. The resting membrane potential during recordings was −71.5 ± 1.5 mV.

To determine if PnTXs modified the sensitivity of nAChR to extracellularly applied ACh, an intracellular recording microelectrode was impaled, as close as possible to the endplate area, so that mEPPs with fast time course could be recorded. Then, an iontophoretic micropipette filled with ACh was placed extracellularly in very close contact with the muscle membrane, and to the intracellular microelectrode in the endplate area. Current pulses of 1 ms duration at constant intensity were applied to the pipette to give an ACh potential of 5–8 mV in amplitude (Figure 9, inset). After stable ACh potential recordings, EDL preparations were perfused with 100 nM PnTX-A, and constant ACh pulses were delivered by iontophoresis each 20 or 30 s. In the presence of PnTX-A, the amplitude of the ACh potential decreased in a time-dependent manner so after about 10–14 min (n = 3), no response could be evoked by ACh, as shown in the graph of Figure 9. This result clearly indicates that PnTX-A blocks the muscle-type α1_2_β1δε nAChR subtype.

Additional experiments were performed to determine whether the spiroimine component in PnTX-A is essential for the blocking action of EPP amplitudes. For this, experiments were performed with the synthetic PnTX-AK that includes an open form of the imine ring A. As shown in Figure 7a,b, PnTX-AK had no action on nerve-evoked EPP amplitude at concentrations at which PnTX-A exerted 80% blockade of EPP amplitude. Similarly, no significant effect of PnTX-AK (5.44 nmol/kg of mouse) was detected on the CMAP recorded from anesthetized mice in vivo (n = 3, data not shown). These data indicate that the spiroimine component constitutes a critical structural factor for the action of PnTXs in vitro and in vivo.

### 2.4. Computational Modeling of PnTX-A and G Interactions with the Nicotinic Acetylcholine Receptor

**Figure 10:**
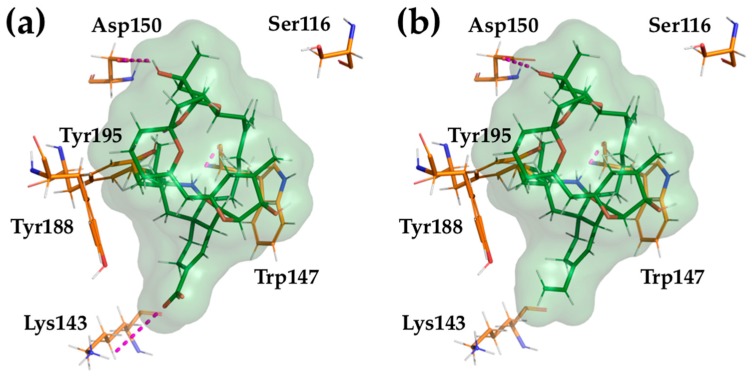
Protein-ligand interactions in the docking complexes of PnTX-A (**a**) and PnTX-G (**b**) with the mouse nAChR at the α1-ε interface. Only amino acids interacting through hydrogen bonds with the ligand or involved in toxin’s subtype selectivity, and in the sequence alignment, are shown. The numbering of amino acid residues is the same as in [31].

Molecular docking calculations of PnTX-A and G were carried out at the interface α1-ε of mouse muscular nAChRs. Overall, the binding modes observed are similar to those described previously for PnTX-A at the interface α1-δ of the *Torpedo* nAChR [31]. There are, however, significant differences in the protein-ligand interactions that are described in detail below (Figure 10).

In all cases, the hydrogen bond between the spiroimine group and the backbone oxygen of α1-Trp147, which is the signature of these classes of toxins, is conserved. The hydrogen bond between the carboxylate group of PnTX-A and the side chain of α1-Lys143 is conserved, but is not possible for PnTX-G, in which the carboxylate is replaced by a vinyl group. Another conserved interaction, observed for both PnTX-A and G, is made by the side chain of α1-Tyr195, which established a hydrogen bond with the oxygen from the tetrahydrofuran ring. The α1-Asp193 in *Torpedo* nAChRs corresponds to α1-Thr193 in mouse nAChRs, which is too short to interact with the hydroxyl group in position 15 of PnTX-A and G. Instead, we observe a hydrogen bond of this hydroxyl with the side chain of α1-Asp150.

Overall, our *in silico* study of the interactions between PnTX-A and G and the interface α1-ε of mouse muscle-type nAChR highlights several important differences, but also common features, compared with the interaction of PnTX-A with *Torpedo* nAChRs that was previously described [31]. Thus, these various interaction patterns allow a fine tuning for the affinity and the specificity of different ligands against the nAChRs subtypes and their binding interfaces.

## 3. Discussion

The present experimental results demonstrate, for the first time using a multimodal minimally-invasive in vivo electrophysiological approach, that synthetic PnTX-A and G, when injected locally to anesthetized mice, caused a time- and dose-dependent decrease of the CMAP recorded from the tail muscle in response to motor nerve stimulation. The CMAP block produced by both toxins in vivo was reversible within 6–8 h. Compared to other cyclic imine toxins, PnTX-A and G had a similar potency in vivo than GYM-A [36], but were less potent than 13-SPX-C [36] and 20-meSPX-G [37].

In vitro, PnTX-A and G blocked, in a reversible manner, nerve-evoked muscle contraction in the mouse peroneal nerve-EDL muscle preparation without affecting directly-elicited twitch or tetanic contractions. This is consistent with previous work done with other cyclic imine toxins like GYM-A [38], 13-SPX-C, and 13,19-didesmethyl spirolide C [39]. These results indicate that both PnTX-A and G affect neuromuscular transmission, but have no action on the contractile machinery of muscle fibers. This is in agreement with observations showing that 40 mM K^+^-induced muscle contractures were unaffected after treatment with PnTX-E, F and G [35]. A rank order of potency of PnTX-F > PnTX-G > PnTX-E has been reported in vitro on nerve-evoked twitch response in rat phrenic nerve-hemidiaphragm muscle [35]. In the present study, a rank order of potency PnTX-G > PnTX-A was obtained in the mouse peroneal nerve-EDL muscle. It is not possible to compare former and here-presented data on PnTX potency, since they have been done in different species and also in different muscles, and is well known that the diaphragm is more resistant to the action of neuromuscular blocking agents than limb muscles [41]. 

In contrast to previous studies with PnTX-E, F and G, extracted from dinoflagellates, which were performed in rat phrenic-cut-hemidiaphragm muscle, with an extracellular medium having a reduced K^+^ concentration [35]. Present intracellular recordings were performed in normal mouse EDL and FDB nerve-muscle preparations, equilibrated in normal Krebs-Ringer solution, and in some experiments a selective blocker of voltage-gated muscle Na^+^ channel was used for blocking muscle contraction. Analyses of the action of both PnTX-A and PnTX–G on neuromuscular transmission at single junctions of EDL and FDB revealed that both phycotoxins blocked the EPP amplitude. The block of EPP amplitude to subthreshold levels for action potential generation, at single junction, can explain the dose-dependent reduction in the CMAP observed in vivo by extracellular recordings in the caudal muscle. The reduction of the amplitude of spontaneous mEPP, without significant change in the mEPP frequency, and the block of full-sized EPPs and ACh-evoked potentials strongly indicate that PnTX-A and G exert a selective action at the postsynaptic level of the neuromuscular junction by blocking the interaction between ACh and the α1_2_β1δε nAChR. Whether PnTXs have a presynaptic action was not directly investigated in our study. However, data obtained by measuring mEPP frequency, when there was about 50% block of mEPP amplitude, strongly suggests that PnTXs do not have a pre-synaptic action that would modify the quantal ACh release rate. Insight into the interaction between PnTX-A and PnTX-G and the muscle-type α1_2_β1γδ nAChR was previously obtained in competition binding studies using [^125^I] α-bungarotoxin as a tracer. In those studies, both phycotoxins totally displaced, in a concentration-dependent the radiotracer [31,32]. Thus, PnTXs on the muscle-type α1_2_β1γδ nAChR act as competitive antagonists. That 3,4-DAP was able to reverse the postsynaptic neuromuscular block produced by PnTX-A is an indication that PnTX-A may act also as a competitive antagonist on the α1_2_β1δε nAChR. 3,4-DAP is known to block a fast K^+^ current in motor nerve terminals, to greatly increase nerve-evoked quantal ACh release and to antagonize the competitive action of d-tubocurarine on the α1_2_β1δε nAChR [42,43,44]. Taken together, the functional data obtained in the present study indicate that PnTX-A and PnTX-G block mouse neuromuscular transmission, which can explain the muscle paralysis and death via respiratory depression when administered in vivo in toxicity studies [6,23,30]. 

## 4. Materials and Methods

### 4.1. Toxins and Chemicals

PnTX-A, G and AK were synthesized, as previously reported [31]. The purity of PnTXs and analogue was over 98%, as judged by NMR data and HPLC performed for final purification (Supplementary data). The µ-conotoxin GIIIB was obtained from Alomone Labs (Alomone Labs, Jerusalem, Israel). All chemicals, including ACh hydrochloride and 3,4-DAP, were purchased from Sigma-Aldrich (Saint Quentin Fallavier, France).

The in vivo experiments were performed using a stock solution of PBS (1X, 100 µL) added to 1% methanol (vehicle) and either 28 µM PnTX-A or 20 µM PnTX-G. PnTX-A was studied at doses of 0.54–5.44 nmol/kg of mouse. PnTX-G was studied at doses of 1.60–3.20 nmol/kg of mouse.

### 4.2. Animals

Adult male and female Swiss mice (*Mus musculus*, 2–5 months of age and 23–28 g of body weight) were purchased from Janvier Elevage (Le Genest-Saint-Isle, France), and acclimatized at the CEA animal facility for at least 48 h before experiments. Live animals were treated according to the European Community guidelines for laboratory animal handling and to the guidelines established by the French Council on animal care “Guide for the Care and Use of Laboratory Animals” (EEC86/609 Council Directive – Decree 2001-131). In particular, they were housed four- to six-wise in cages with environmental enrichment, in a room with constant temperature and a standard light cycle of 12-h light/12-h darkness, and had free access to water and food.

All experimental procedures on mice were approved by the Animal Ethics Committee of the CEA (project 17_088 authorized to E.B.) and by the French General Directorate for Research and Innovation (project APAFIS#2671-2015110915123958v4 authorized to E.B.).

### 4.3. Recordings from the Neuromuscular System of Anesthetized Mice In Vivo

The multimodal excitability properties of the mouse neuromuscular system were assessed in vivo on females [weighting 26.4 ± 1.4 g (n = 26)], under isoflurane (AErrane, Baxter S.A., Lessines, Belgium) anesthesia, by means of minimally-invasive electrophysiological methods using the Qtrac^©^ software (Bostock H., Institute of Neurology, London, UK), as previously described [45]. Briefly, the CMAP was recorded using fine needle electrodes inserted into the tail muscle and connected to an amplifier (Disa EMG 14C13), in response to electrical stimulations delivered to the caudal motor nerve by two stimulators (A395, World Precision Instruments, Sarasota, FL, USA) via surface electrodes. To study the underlying mode of action of PnTX-A and G, intramuscular injections (5-µL maximal volume) of PBS containing 0.1–1% methanol and various doses of PnTX-A (from 0.54 to 5.44 nmol/kg of mouse) or PnTX-G (1.60 and 3.20 nmol/kg of mouse) were delivered with a 10-µL micro-syringe at the base of the mouse tail, between stimulation and ground electrodes. Similar injections (5 µL) were also done with PBS containing only 1% methanol to test an eventual effect of the vehicle associated with the highest dose of toxins studied.

On-line recordings were initiated approximately 10 min before a given injection to observe the effects over time of PnTX-A, PnTX-G, and/or methanol on selected excitability parameters such as the CMAP amplitude and excitability threshold, registered continuously. To further identify the toxin underlying mechanism of action and duration of effects, five different excitability tests (stimulus-response, strength-duration and current-threshold relationships, as well as threshold electrotonus and recovery cycle) [46] were performed together before and various times (from 45 min to 12 h) after a given injection. As a whole, more than thirty parameters were determined from these five different excitability tests and analyzed, providing additional and complementary information on the functional status of ion channels, receptors and electrogenic pumps, as well as on membrane properties of the neuromuscular system [47,48].

### 4.4. Recordings from Isolated Mouse Nerve-muscle Preparations In Vitro

Male and female mice were anesthetized with isoflurane inhalation before being euthanized by dislocation of the cervical vertebrae. In vitro assays on isometric twitch tension were performed on isolated EDL muscle, and those on synaptic transmission on isolated EDL and flexor digitorum brevis (FDB) muscles. Removal of muscles and dissections were performed within 20 min in an oxygenated Krebs-Ringer solution of the following composition (in mM): NaCl 150, KCl 5, CaCl_2_ 2, MgCl_2_ 1, glucose 11, and HEPES 5 (pH 7.4).

For isometric twitch tension measurements, the EDL muscle with its attached peroneal nerve was carefully dissected and mounted in a silicone-lined bath filled with an oxygenated Krebs-Ringer solution. One of the EDL tendons was securely anchored onto the silicone-coated bath, while the other was attached via an adjustable stainless-steel hook to an isometric force transducer (FT03; Grass Instruments, West Warwick, RI, USA). Muscle twitches and tetanic contractions were evoked either by stimulating the motor nerve via a suction microelectrode (adapted to the diameter of the nerve), with supramaximal current pulses of 0.15 ms duration, at frequencies indicated in the text, delivered by the isolation unit of a stimulator (S-44 Grass Instruments) or by direct electrical stimulation through an electrode assembly build up in the silicone-coated bath and placed at a short distance along the length of the muscle. For each preparation investigated, the resting tension was adjusted with a mobile micrometer stage which allowed incremental variations of the muscle length in order to obtain maximal contractile responses. Signals from the isometric transducer were amplified, collected, and digitized with the aid of a computer equipped with a Digidata-1322A A/D interface board (Axon Instruments, Molecular Devices, Sunnyvale, CA, USA). Data acquisition and analysis were performed with the WinWCP v3.9.6 software program (John Dempster, University of Strathclyde, Scotland). Experiments were performed at constant room temperature (22 °C).

For synaptic transmission measurements, either EDL or FDB nerve-muscle preparations were dissected and mounted in silicone-lined recording chambers with stainless mini-pins. The motor nerve was stimulated with a suction electrode, with pulses of 0.1 ms duration and supramaximal voltage (typically 3–8 V). Both internal and external platinum wires of the suction electrode were connected to the isolation unit of a stimulator (S-44 Grass Instruments). Intracellular recordings were performed with 1.2 mm (external diameter) borosilicate glass capillaries containing internal glass filaments (type GC120f; Clark Electromedical Instruments, Pangbourne, UK) pulled on a P-1000 puller (Sutter Instrument Company, Novato, CA, USA) and having a resistance of 6–12 MΩ when backfilled with a 3 M KCl solution. Conventional intracellular techniques were used to record the resting membrane potential, end-plate potential (EPP) and miniature end-plate potential (mEPP) with an Axoclamp-2B amplifier. “Floating” microelectrodes were made as described [49] and used to record action potential in contracting muscles. In some experiments, nerve-muscle preparations were incubated for 30–45 min with µ-conotoxin GIIIB (1.0–1.6 µM) to block muscle contraction upon nerve stimulation [40]. After muscle fiber impalement, synaptic potentials were digitized using a Digidata-1322A A/D interface board (Axon Instruments, Sunnyvale, CA, USA). For iontophoretic ACh application, high resistance pipettes (100–150 MΩ) made with borosilicate glass and filled with 1 M ACh hydrochloride were used. ACh efflux was induced by cationic-current pulses (1 ms duration) using a constant current generator that allowed the use of breaking currents to prevent spontaneous ACh efflux and avoid nAChR desensitization.

### 4.5. Data and Statistical Analyses

Sigmoid non-linear regressions through data points (correlation coefficient = r^2^) were used to calculate theoretical dose/concentration-response curves, according to the Hill equation (GraphPad Prism version 5):

Rt/Rc = 1/[1 + ([toxin]/X) ^nH^], (1)

where Rt/Rc is the response recorded in the presence of a given toxin (Rt) and expressed as percentage of the value obtained in absence of toxin (Rc), [toxin] is the toxin concentration, X is the toxin dose (ID_50_) or concentration (IC_50_) necessary to inhibit 50% of the response, and nH is the Hill number.

EPP amplitudes (exceeding 3 mV) were corrected for non-linear summation according to McLachlan and Martin 1981 [50]. In the present study we have used an “f“ factor of 0.8, and an equilibrium potential for ACh of 0 mV in the calculation of EPP amplitudes in mouse EDL neuromuscular preparations.

Data are presented as means ± standard deviations (S.D.) of n different experiments. Differences between values were tested using the parametric two-tailed Student’s *t*-test (either paired samples for comparison within a single population or unpaired samples for comparison between two independent populations), and the one- or two-way analysis of variance (ANOVA for comparison between the means of independent populations) or the non-parametric Mann-Whitney U-test, depending on the equality of variances estimated using the Lilliefors’ test. Differences were considered statistically significant when *P* < 0.05.

### 4.6. Molecular Modeling

A homology model of the extracellular domain of mouse (α1)_2_β1δε nAChRs subtype was constructed using Modeller [51], and the *Aplysia californica* acetylcholine binding protein (AChBP) crystal structure as template (Protein Data Bank code 2WZY) [52]. Three-dimensional structures of PnTX-A and G were generated using Corina 3.6 (Molecular Networks GmbH, Erlangen, Germany, 2018). The docking procedure was carried out at the interface α1-ε in a similar manner as previously described for PnTX-A at the interface α1-δ of *Torpedo* nAChRs [31], using Gold (Cambridge Crystallographic Data Centre, Cambridge, UK) and the GoldScore scoring function. The binding site, defined as a 20 Å radius sphere, was centred on the backbone oxygen atom of Trp147. All other parameters had default values.

The receptor-ligand complexes images were produced using Pymol 2.2.0 (Schrödinger, LLC, New York, NY, USA, 2019).

## 5. Conclusions

The study of multimodal excitability properties of mouse neuromuscular system in vivo revealed that PnTX-G is as efficient as PnTX-A to produce a reversible inhibition of CMAPs recorded from the tail muscle, without any significant modification of other excitability parameters. The block of muscle nAChR by PnTX-A and G, as shown in vitro, reduced the amplitude of EPPs to subthreshold levels for action potential generation in muscle fibers and explains the reduction of CMAP in vivo. Synaptic transmission in vitro was strongly impaired by both PnTX-A and G, since both phycotoxins blocked, in a reversible manner, the interaction of ACh quanta with endplate nAChR. Thus, PnTX-A and G are potent muscle-type nAChR antagonists. 3,4-DAP reversed the post-synaptic blockade produced by PnTX-A. Modeling studies revealed the molecular determinants responsible for the interaction of PnTXs with the muscle-type nAChR. 

## Figures and Tables

**Table 1:** ID_50_ values (in nmol/kg of mouse) of some cyclic imine toxins, determined from CMAP maximal amplitude recorded from mouse tail muscle in vivo.

ID_50_	PnTX-A	PnTX-G	GYM-A	13-SPX-C	20-meSPX-G
**Mean**	3.080	2.830	3.474	0.009	0.002
**S.D.**	0.203	0.090	0.166	0.001	0.000
**n (mice)**	18	8	15	19	26
**Reference**	Present study	Present study	[36]	[36]	[37]

**Table 2:** Excitability parameters (means ± S.D.) from tail muscle recordings of mice in vivo, before (control, n = 15 mice) and approximately 45 min after injection of PBS containing methanol (1%) and PnTX-A (5.44 nmol/kg of mouse, n = 8 mice).

Test ^1^	Excitability Parameter ^2^	Control	PnTX-A	*P*
T1	Peak response (mV)	3.299 ± 1.140	1.847 ± 1.370	0.050 *
	Latency (ms)	3.359 ± 0.065	3.349 ± 0.106	0.893
	Stimulus (mA) for 50% max response	0.481 ± 1.060	0.479 ± 1.130	0.927
	Stimulus-response slope	3.004 ± 1.180	2.650 ± 1.310	0.743
T2	Resting slope	0.638 ± 0.077	0.916 ± 0.257	0.182
	Minimum slope	0.230 ± 0.013	0.218 ± 0.022	0.703
	Hyperpolarizing slope	0.536 ± 0.071	0.490 ± 0.074	0.757
T3	Strength-duration time constant (ms)	0.377 ± 0.035	0.316 ± 0.036	0.274
	Rheobase (mA)	0.333 ± 1.070	0.327 ± 1.130	0.861
T4	TEd (10–20 ms)	47.680 ± 2.570	45.250 ± 4.930	0.700
	TEd (peak)	48.640 ± 2.320	45.240 ± 4.110	0.555
	TEd (40–60 ms)	39.440 ± 3.000	38.080 ± 1.980	0.826
	TEd (90–100 ms)	36.000 ± 3.010	35.230 ± 1.810	0.878
	TEd (accommodation)	12.590 ± 1.230	10.170 ± 2.890	0.441
	TEd (undershoot)	−11.820 ± 1.120	−5.188 ± 0.512	0.019 *
	TEh (10–20 ms)	−97.600 ± 4.780	−86.230 ± 2.660	0.318
	TEh (20–40 ms)	−132.000 ± 8.430	−123.900 ± 7.160	0.686
	TEh (90–100 ms)	−159.20 ± 12.60	−150.300 ± 4.910	0.753
	TEh (overshoot)	13.350 ± 1.460	7.238 ± 1.450	0.079
T5	Relative refractory period (ms)	2.349 ± 1.090	2.532 ± 1.100	0.632
	Superexcitability (%)	−7.596 ± 1.490	−9.049 ± 1.970	0.600
	Subexcitability (%)	3.844 ± 0.708	3.316 ± 1.160	0.697

^1^ T1 = Stimulus-response relationship, T2 = Current-threshold relationship (informed on accommodation capacity to depolarizing and hyperpolarizing currents), T3 = Strength-duration relationship (informed on nodal membrane potential), T4 = Threshold electrotonus and T5 = Recovery cycle. ^2^ TEd = Threshold electrotonus from depolarizing currents and TEh = Threshold electrotonus from hyperpolarizing currents. *: Significant difference compared to control.

## Supplementary Materials

The following are available online at https://www.mdpi.com/1660-3397/17/5/306/s1, Figure S1: 800 MHz 1H (a) and 200 MHz 13C (b) NMR spectra of PnTX-A recorded as a solution in CD3OD, Figure S2: 800 MHz ^1^H (a) and 200 MHz ^13^C (b) NMR spectra of PnTX-G trifluoroaceate recorded as a solution in CD_3_OD, Figure S3: 500 MHz ^1^H (a) and 125 MHz ^13^C (b) NMR spectra of PnTX-AK recorded as a solution in CD_3_OD.
