# Supplementary material for: Synthetic Pinnatoxins A and G Reversibly Block Mouse Skeletal Neuromuscular Transmission In Vivo and In Vitro
Source: Mar Drugs. 2019 May 24;17(5):306. doi: 10.3390/md17050306 (PMC6562580; doi:10.3390/md17050306)
Supplement: Supplementary file 1 [file marinedrugs-17-00306-s001.pdf]

marine drugs

MDPI

"Synthetic Pinnatoxins A and G Reversibly Block Mouse Skeletal Neuromuscular Transmission *in vivo* and *in vitro*" by Evelyne Benoit, Aurélie Couesnon, Jiri Lindovsky, Bogdan I. Iorga, Rómulo Aráoz, Denis Servent, Armen Zakarian and Jordi Molgó

Supplementary data

Copies of ¹H and ¹³C NMR spectra for PnTX-A, PnTX-G and PnTX-AK

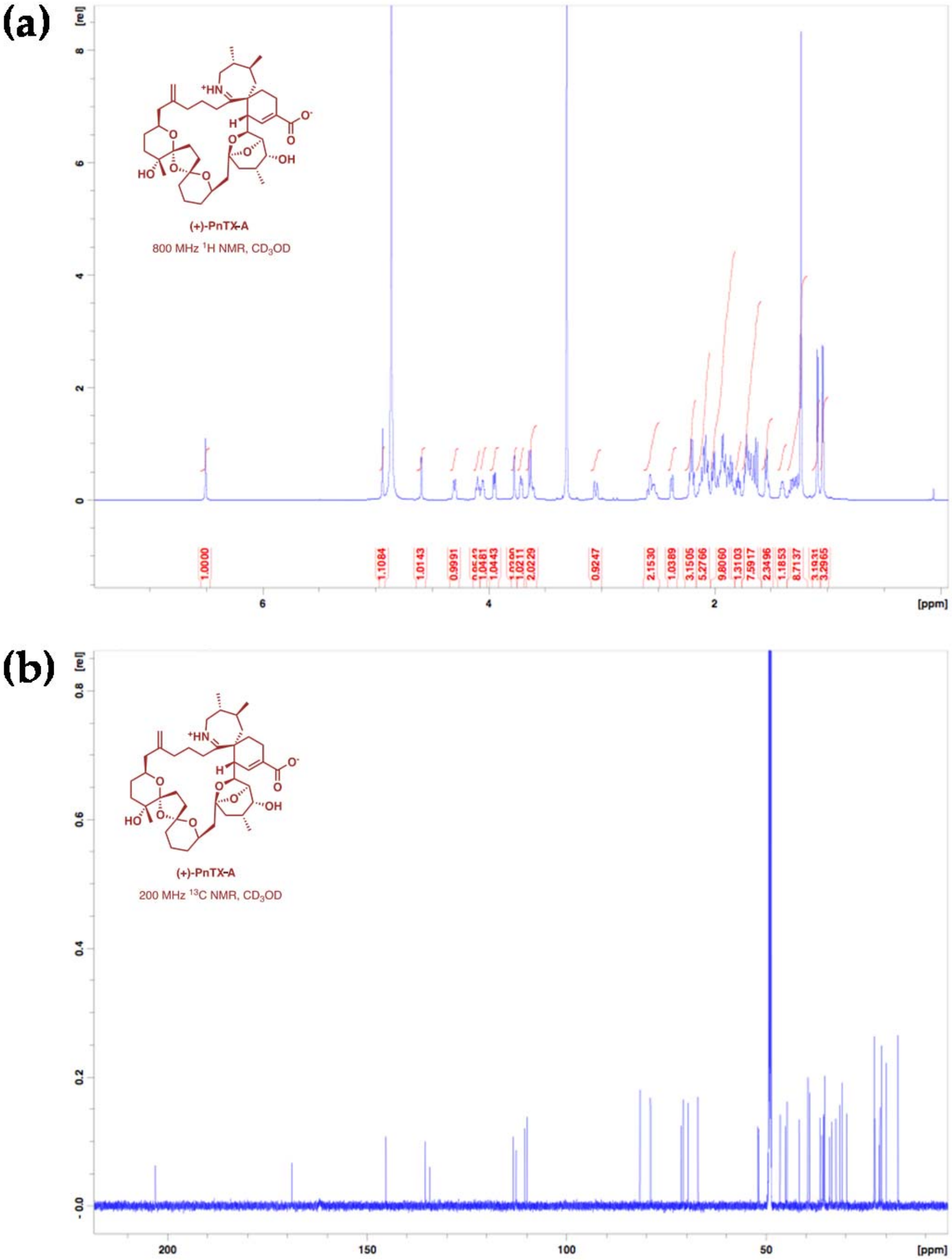

Supplementary Figure 1. 800 MHz ¹H (**a**) and 200 MHz ¹³C (**b**) NMR spectra of PnTX-A recorded as a solution in CD₃OD.

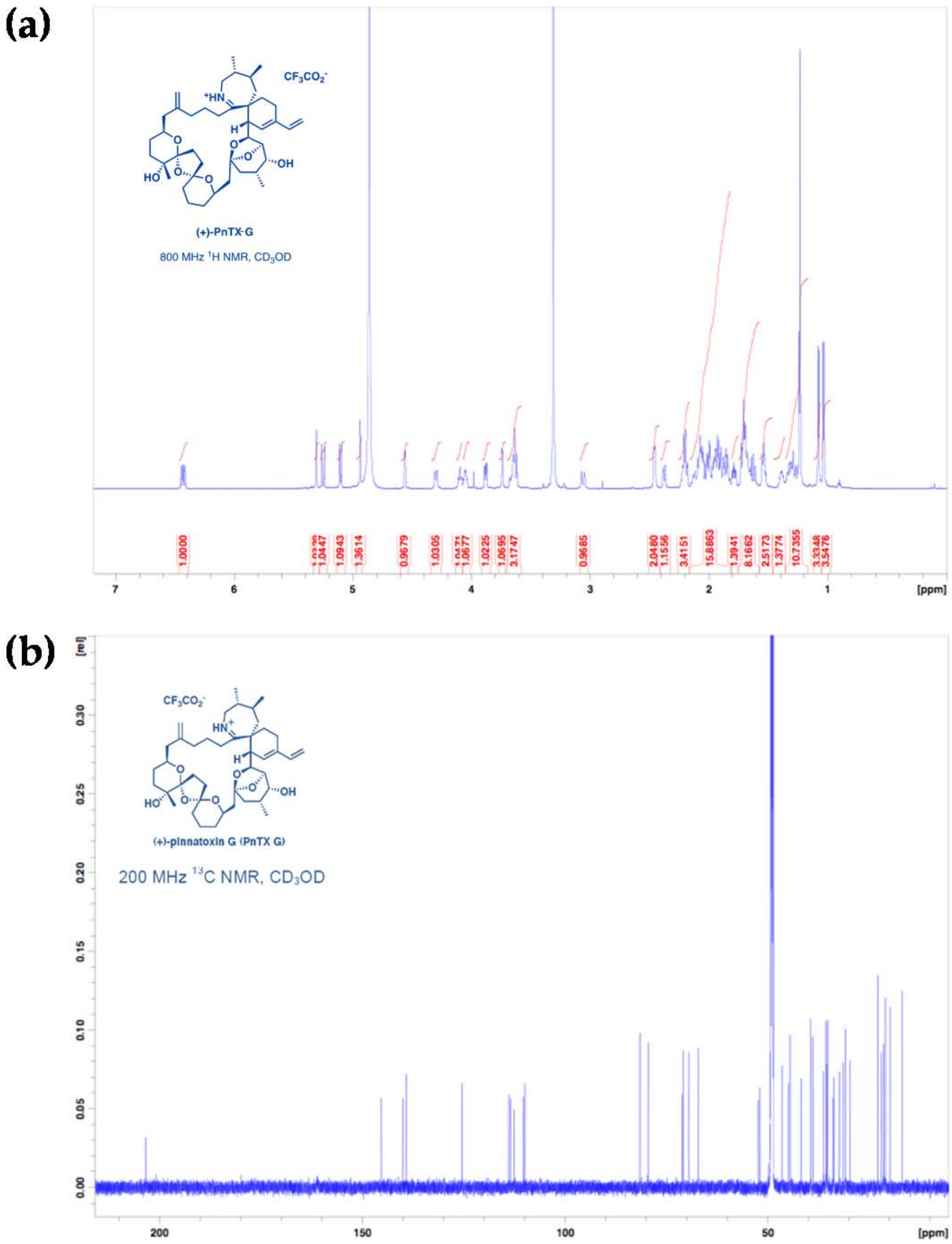

Supplementary Figure 2. 800 MHz ^{1}H (**a**) and 200 MHz ^{13}C (**b**) NMR spectra of PnTX-G trifluoroaceate recorded as a solution in CD_3OD.

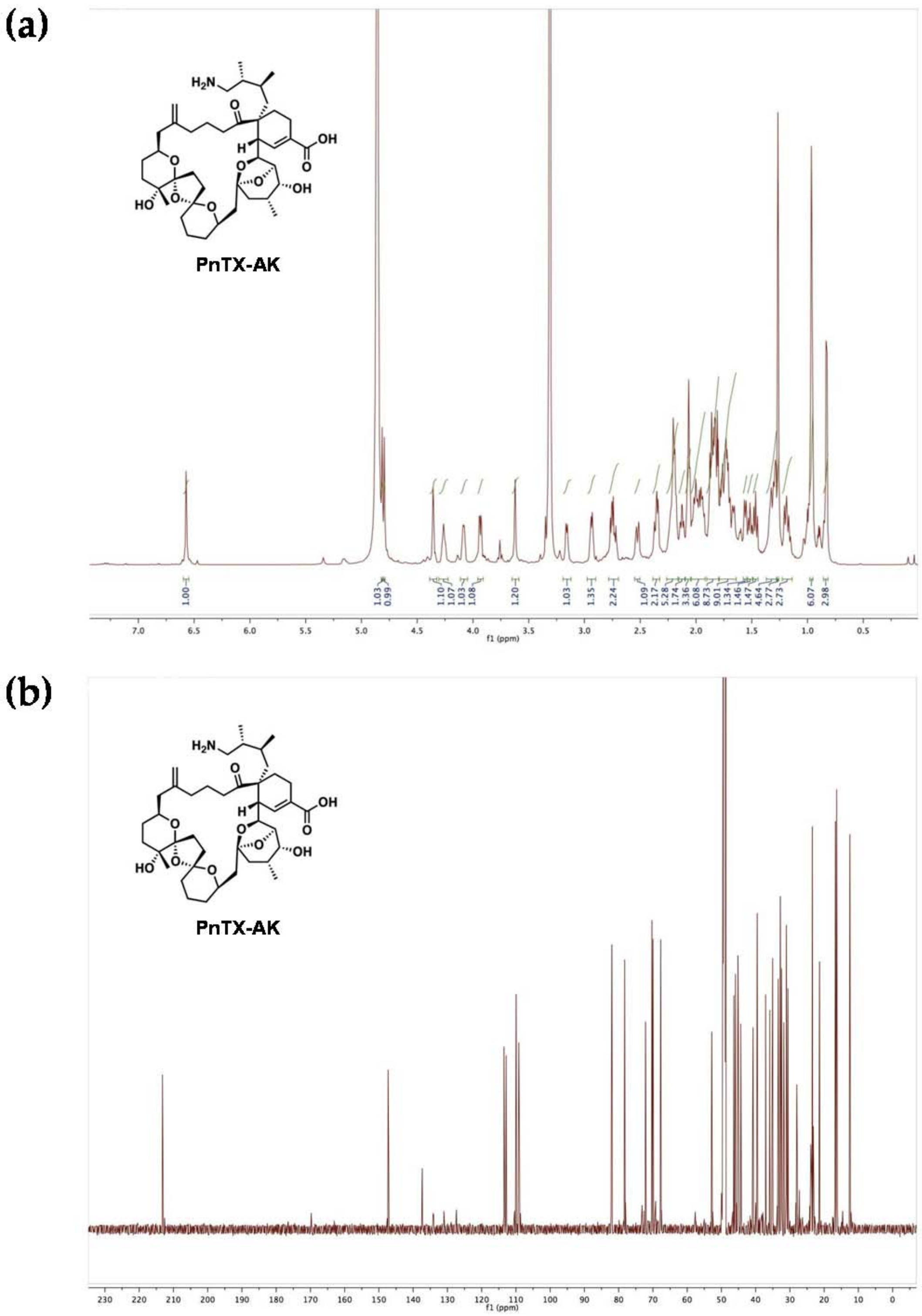

Supplementary Figure 3. 500 MHz ^{1}H (**a**) and 125 MHz ^{13}C (**b**) NMR spectra of PnTX-AK recorded as a solution in CD_3OD.

© 2019 by the authors. Submitted for possible open access publication under the terms and conditions of the Creative Commons Attribution (CC BY) license (http://creativecommons.org/licenses/by/4.0/).
